# Predicted Molecular Effects of Sequence Variants Link to System Level of Disease

**DOI:** 10.1371/journal.pcbi.1005047

**Published:** 2016-08-18

**Authors:** Jonas Reeb, Maximilian Hecht, Yannick Mahlich, Yana Bromberg, Burkhard Rost

**Affiliations:** 1 Department of Informatics, Bioinformatics & Computational Biology—i12, Technische Universität München, Garching/Munich, Germany; 2 TUM Graduate School, Center of Doctoral Studies in Informatics and its Applications (CeDoSIA), Technische Universität München, Garching, Germany; 3 Department of Biochemistry and Microbiology, Rutgers University, New Brunswick, New Jersey, United States of America; 4 Institute for Advanced Study (TUM-IAS), Garching/Munich, Germany; 5 Institute for Food and Plant Sciences WZW, Technische Universität München, Weihenstephan, Freising, Germany; Johns Hopkins University, UNITED STATES

## Abstract

Developments in experimental and computational biology are advancing our understanding of how protein sequence variation impacts molecular protein function. However, the leap from the micro level of molecular function to the macro level of the whole organism, *e*.*g*. disease, remains barred. Here, we present new results emphasizing earlier work that suggested some links from molecular function to disease. We focused on non-synonymous single nucleotide variants, also referred to as single amino acid variants (SAVs). Building upon OMIA (Online Mendelian Inheritance in Animals), we introduced a curated set of 117 disease-causing SAVs in animals. Methods optimized to capture effects upon molecular function often correctly predict human (OMIM) and animal (OMIA) Mendelian disease-causing variants. We also predicted effects of human disease-causing variants in the mouse model, *i*.*e*. we put OMIM SAVs into mouse orthologs. Overall, fewer variants were predicted with effect in the model organism than in the original organism. Our results, along with other recent studies, demonstrate that predictions of molecular effects capture some important aspects of disease. Thus, *in silico* methods focusing on the micro level of molecular function can help to understand the macro system level of disease.

## Author Summary

The variations in the genetic sequence between individuals affect the gene-product, *i*.*e*. the protein differently. Some variants have no measurable effect (are neutral), while others affect protein function. Some of those effects are so severe they cause so called monogenic Mendelian diseases, *i*.*e*. diseases triggered by a single letter change. Some *in silico* methods predict the molecular impact of sequence variation. However, both experimental and computational analyses struggle to generalize from the effect upon molecular protein function to the effect upon the organism such as a disease. Here, we confirmed that methods predicting molecular effects correctly capture the type of effects causing Mendelian diseases in human and introduced a data set for animal diseases that was also captured by predictions methods. Predicted effects were less when *in silico* testing human variants in an animal model (here mouse). This is important to know because “mouse models” are common to study human diseases. Overall, we provided some evidence for a link between the molecular level and some type of disease.

## Introduction

Protein sequences span three orders of magnitude in their lengths (30-30k residues). Aspects of molecular function are often captured by ‘sub-units’, *e*.*g*. by domains or domain-like fragments [1,2] that are, on average, about 100 residues long [3,4]. The variation of a single amino acid (SAV) can change the function of a multi-domain protein and many changes in molecular function lead to disease. In fact, OMIM, the database of Online Mendelian Inheritance in Man [5], archives thousands of SAVs that cause Mendelian diseases. On the other hand, databases such as the Protein Mutant Database (PMD) catalogue tens of thousands SAVs altering molecular function; many of those have not been observed to cause a phenotype on the level of the organism. Sequencing everyone on this globe, will we observe almost all possible SAVs? The answer remains subject for speculation. Obvious exceptions include embryonically lethal variants and not all variants will occur in germ lines.

Deep mutational scanning studies that change every residue in a protein to all non-native amino acids suggest a conundrum: for almost every position (each residue) both neutral and effect SAVs exist [6–8], *i*.*e*. most residue positions are at the same time sensitive and robust to variants. A variety of computational methods predict the effect of SAVs. Although most methods have many goals, we can simplify by distinguishing methods that focus more on predicting the effect of SAVs upon (Mendelian) disease [9–15] and upon molecular function or structure [16–20]. *In silico* methods focusing on molecular function [21,22] correlate more with experimental deep mutational scans than those focusing on disease [8,23].

The “micro” perspective of molecular function is often probed through *in vitro* assays of proteins or cells, while *in vivo* screens often focus on observing the “macro” level through the impact upon the entire organism or system, *e*.*g*. in form of a disease phenotype. Molecular impact does not directly correspond to system impact, *i*.*e*. functional effects of variants usually do not directly explain diseases. Relating the two levels of variant effects is of utmost importance, for example to understand diseases and to develop treatments. Successful drugs often mechanistically bridge this gap: the molecular agent (drug) affects the organism/system (disease).

Here, we show a few links that suggest how molecular effect predictions can capture some aspects of diseases. Our findings are largely based on a manually curated set of variants (SAVs) from OMIA (Online Mendelian Inheritance in Animals), a database cataloging expert curated monogenic diseases in animals and their relevant variants [24]. Methods focusing on the molecular impact of variants predict disease-causing variants in animals and human (taken from OMIM [5]). We also addressed the question how prediction methods behave for model systems, *e*.*g*. by predicting variants in mice to study human diseases. The latter analysis might be particularly relevant in light of a recent discussion about the validity of using mouse models [25,26].

## Results and Discussion

### OMIM variants predicted to have strong effect

**Fig 1:**
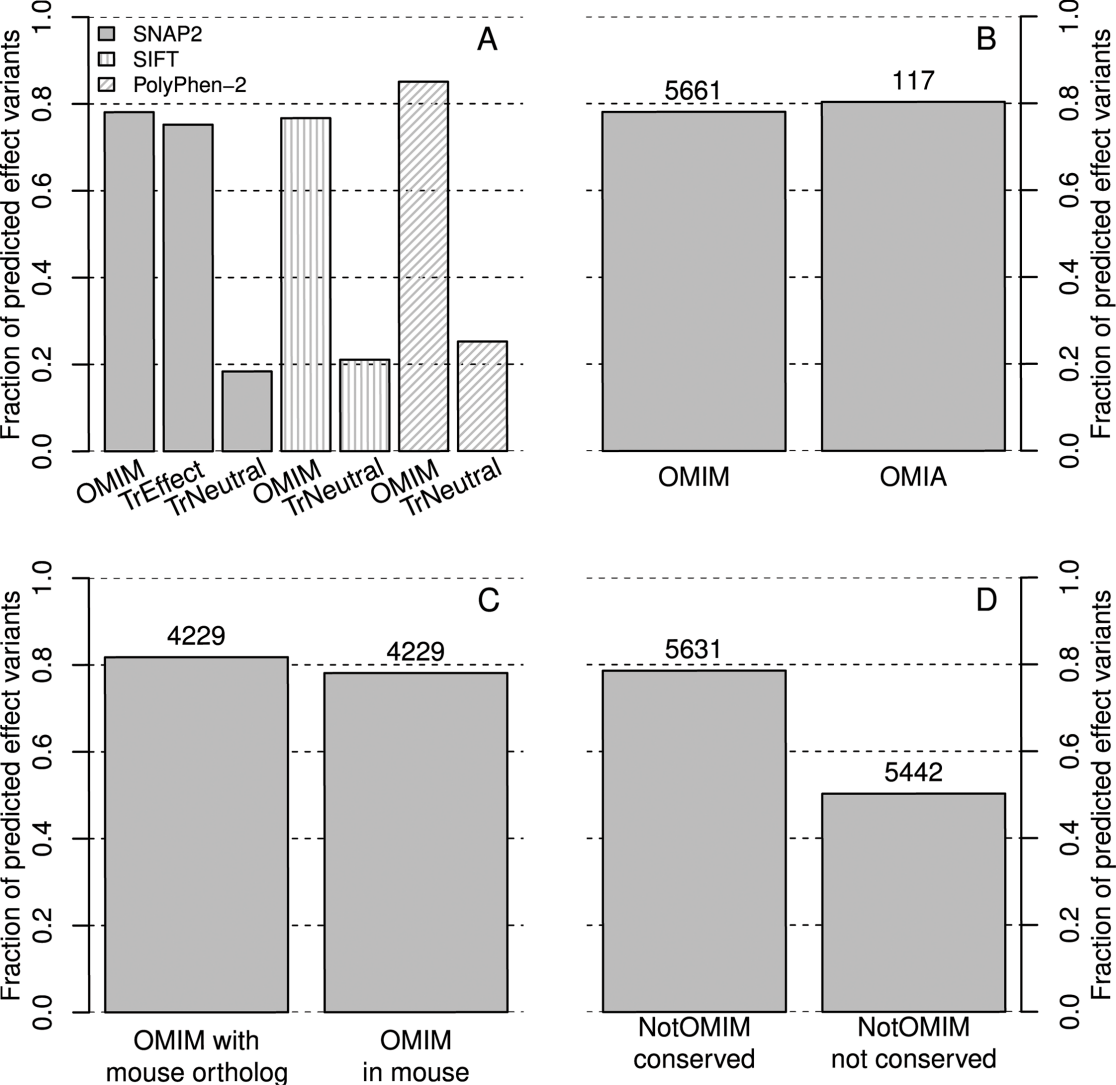
Predictions of SAV effects upon function and disease across species. The numbers above bars give the number of SAVs in the set. **A**: Three methods (SNAP2 [16], SIFT [27], PolyPhen-2 [12]) predicted SAV effects upon molecular function (TrEffect/TrNeutral) and upon disease (OMIM). Exclusively for this panel SNAP2 was trained without using disease SAVs from OMIM [5] or HumVar [28]. The SNAP2 version trained exclusively on molecular function clearly captured aspects of OMIM-disease SAVs (leftmost bar OMIM higher than 2^nd^ to the left TrEffect). TrNeutral was the SNAP2 training set of variants without effect. Comparing the bars for TrNeutral and OMIM for each method pointed to differential thresholds: Polyphen-2 correctly predicted more effect in OMIM than SNAP2 but also incorrectly predicted more effect in the neutral data, *i*.*e*. simply predicted more effect variants. **B:** OMIM is repeated from A. SNAP2 captured disease signals in humans and animals at similar levels. OMIA contained disease SAVs from animals other than mouse and rat (mostly dog and cattle). **C:** SNAP2 predicted OMIM SAVs with less effect in mouse orthologs than in human. Left bar (*OMIM with mouse ortholog*): SNAP2 predictions for the subset of all 4,229 OMIM SAVs for which we found a mouse ortholog. Right bar (*OMIM in mouse*): SNAP2 predictions when putting the human SAV into the mouse sequence. **D:** Disease variants happen in non-random positions. Left bar (*NotOMIM conserved*): in each protein with an OMIM SAV, we predicted the effect of all SAVs with a level of sequence conservation ≥ that of the OMIM variant. Right bar (*NotOMIM not conserved*): predictions for SAVs in non-OMIM positions with conservation < that of the OMIM SAV. Obviously, OMIM SAVs were very well conserved.

SIFT [27] predicts the impact of variants upon molecular protein function by assessing the disruption of conserved residues. SNAP [17] predicts this impact by considering evolutionary, functional and structural features. Our newer method SNAP2 [16] also trained on disease-causing variants. To avoid the overlap of variant sets used for SNAP2 training and those used in this work, we trained a SNAP2 version, using only variants with impact upon molecular function, *i*.*e*. leaving out all human disease variants from OMIM or HumVar [28] but keeping the variants from PMD. PolyPhen-2 also uses evolutionary and structural features to predict the effect of disease-causing mutations in human [12]. We predicted the effect of disease-causing SAVs from OMIM through PolyPhen-2, SIFT and the re-trained version of SNAP2 (not using disease variants). All three methods predicted very strong functional effects (Fig 1A). PolyPhen-2 predicted the highest fraction (85%) of the OMIM SAVs to have effects, followed by SNAP2 (78%) and SIFT (76%). Monitoring effect predictions for a set of neutral SAVs (TrNeutral), showed that both PolyPhen-2 and SIFT reached higher effect fractions at the expense of more false positives (TrNeutral bars higher): the differences OMIM-TrNeutral were the same between SNAP2 and PolyPhen-2 (60%). Another crucial difference was that the numbers for SNAP2 were derived without using the data used for training, while the results for PolyPhen-2 overlapped substantially with the training data used for that method. Machine learning methods usually perform better on the training than on the testing data. For instance, the SNAP2 version trained with OMIM reached 80% effect predictions for OMIM as opposed to 78% for the version not trained on OMIM.

Another crucial aspect was that SNAP2 predicted its training set of effect SAVs less well than the OMIM SAVs (Fig 1A: TrEffect 75% vs. OMIM 78%). For us, this was the most outstanding example for a new data set outperforming the training set in 23 years of machine learning in biology [29]. The label “disease” seemingly generates more consistent data than experimental measurements of functional disruption.

Previous analyses showed the strength of the molecular effect to correlate with the SNAP score: higher SNAP scores indicate more reliable predictions and stronger effects [17,30]. This implies that *in silico* predictions can accurately sort thousands of variants relevant for some investigation by their likely molecular impact without the need to provide any additional annotations. Thus, the high amount of SNAP2 effect predictions for OMIM variants (Fig 1A: OMIM higher than for TrEffect) suggested very strong effects upon molecular function. For variants associated with Mendelian disease, this result was expected.

### Manually curated OMIA data set

OMIA [24], the database for Online Mendelian Inheritance in Animals, collects expert annotations for monogenic diseases in animals. Mouse and rat data are excluded, as those variants and annotations are available through the specialized databases RGD [31] and MGD [32]. Unfortunately, none of those resources readily provided the data needed for our analysis. Very few of the, *e*.*g*. 600 variants with known disease associations in OMIA, which range from large structural variants to single nucleotide variants and SAVs, were in a machine-readable standard format such as “sequence variant XpositionY causes effect”. Moreover, the protein sequences referenced by the variants remained obfuscated. Several person-months got us from OMIA to a set of just 117 single disease associated variants with matching sequences (Methods, S2 and S3 Tables). Incidentally, we note that OMIA’s value to the genomics, proteomics and health-related research communities might significantly increase if their high-quality manually curated data were readily available to automated analyses across the spectrum of gene- and protein-science. For similar database-related reasons and time constraints, mouse and rat variants could not be included in this analysis. As an additional complication, studies in mouse and rat typically focus on whole gene knockouts rather than on effects of SAVs.

### Slightly more effect for OMIA than for OMIM variants

All methods optimized to predict disease causes, for obvious reasons of data availability and clinical relevance, focus on human variants. In contrast, methods such as SIFT and SNAP2 perform at similar levels for other organisms. Here, we applied SNAP2 to our curated set of OMIA variants (SAVs). Although this data set was small, it was particularly interesting for testing, because those variants had not been available for the training of methods before.

SNAP2 predicted more OMIA variants with effects than in the SNAP2-effect training set (Fig 1A TrEffect 75% vs. Fig 1B OMIA 80%). Additionally, OMIA variants were predicted with slightly higher effect than those from OMIM (Fig 1B: OMIM 78% vs. OMIA 80%). This result suggested Mendelian disease-SAVs to have stronger effect in animals than in human. The simple asymmetry in what is considered a disease in animals and human might explain this observation. For example, non-lethal abnormalities such as variation in hair-growth might be perceived as a human disease, while the equivalent may not be an animal disease worth noting. In fact, the “disease-ness” of hair/fur length differences actually depends on the animal in question; *e*.*g*. the furs of dogs differ between breeds (an intended result of breeding). OMIA is therefore likely to focus on more lethal variants than OMIM and SNAP2 predictions simply mirror this expectation.

### Disease-variants affect the carrier more than other species

When experimental biology builds an animal model for a human disease, disease-causing human variants are introduced into the animal. Can *in silico* methods achieve the same? We took the mutations (SAVs) from OMIM and predicted the effect of the same variant in the mouse homolog (Fig 1C). The disease-causing SAVs from human were predicted with slightly less effect in the mouse model (Fig 1C: left bar higher than right). We might rationalize this observation by arguing that the OMIM SAV has been observed because it had such a strong effect, slight alterations to the sequence might reduce the signal. Although we have some additional evidence supporting this view (S1 Fig), it remains very speculative. OMIM SAVs are by no means random mutations and in 95% of the cases with OMIM SAVs, the amino acid was the same in human and mouse (not unexpected, given the results presented in the next paragraph). Whatever the cause, this effect should be taken into account when creating animal models for human diseases.

### Position of variant more important than its type

We know that the positions of OMIM variants are not random. *In silico*, we can easily introduce OMIM-like variants elsewhere in the protein. For each OMIM variant (XnY, *i*.*e*. amino acid X at residue n mutated to amino acid Y), we have to find another position (m≠n) and *in silico* vary XmY. Then we compare the predicted effect XnY to those predicted for XmY. As we suspect that OMIM SAVs tend to be more conserved within the evolution of protein families than randomly chosen positions in the same protein, we can additionally constrain our analysis by postulating that we find positions *m* such that the conservation of *m* ≥ that for *n* (Fig 1D: *NotOMIM conserved*). We can contrast this to a sampling in which we predict the effect for less well-conserved positions (m conserved < n, Fig 1D: *NotOMIM not conserved*). This seemingly simple scheme opens another complication: we could additionally choose variants of the native amino acid against all other 19 non-native ones (19-non native), or we could restrict our variants to the subset of those variants that are reachable by a single nucleotide variation (SNV-possible). For simplicity, we only reported results for the SNV-possible version of randomly chosen variants. We observed that a randomly chosen SNV-possible amino acid variant at each OMIM position was predicted with slightly lower effect than the original OMIM SAV (S1 Fig: *OMIM_rand* vs. *OMIM*). More importantly, our results confirmed the expected importance of residue conservation: SNAP2 predicted almost the same effect for the OMIM variant as for NotOMIM SAVs of similar conservation (Fig 1B *OMIM* vs. Fig 1D *NotOMIM conserved*). Conversely, replacing the disease variant XnY at all positions m with less conservation (XmY) was predicted with substantially lower effect (Fig 1D: *NotOMIM conserved* vs. *NotOMIM not conserved*). Interestingly, random SNV-possible variants at OMIM or NotOMIM conserved positions were predicted with an equal number of effect variants (S1 Fig).

We further applied a version of SNAP2 that did not use conservation (*i*.*e*. alignments) as input but was otherwise trained as the default version. This alignment-free version predicted the same trend, but with significantly reduced difference between predicted effect at OMIM and NotOMIM positions (S2 and S3 Figs). Repeating the above analyses for the OMIA set produced similar results (S4–S7 Figs).

The strong dependence of results on conservation suggested that predicting disease-causing variants would only require the definition of a single threshold, *i*.*e*. predict variant as disease if the conservation at its position is above an empirically chosen value. However, we sampled a different conservation threshold for each protein by picking the level of conservation equal to or higher than that observed for each OMIM/OMIA variant. Accordingly, a simple method that predicts every SNV-possible SAV at positions above a single conservation threshold as having an effect, would over-predict effect substantially (S1 Fig, S4 and S6 Figs, S8 Fig).

### Variants with known experimental observations might be biased

SIFT and SNAP2 were optimized on molecular effect variants, PolyPhen-2 [12] on disease variants. Nevertheless, the three agreed on 68% of the variants with known experimental molecular effects [16]. In predicting the effect on molecular function, SNAP2 performed best for difficult variants [16], *i*.*e*. those that were predicted differently by two methods (as effect by one, as neutral by the other). Most relevant and available experimental results have been used for method development. Do computational methods inherit a bias from the experimental data?

We can address the question about bias in the experimental data through comprehensive *in silico* mutagenesis [33], *i*.*e*. by predicting the effect of all possible SAVs; such studies are also referred to as the complete *mutability landscape* [21]. There are two approaches for such a complete mutagenesis: 19 non-native SAVs (large-scale *in silico* mutagenesis), or SNV-possible SAVs. The second approach produces a subset of the first with different statistical features [30]. The first solution furthers our understanding of protein function in the context of its mutability landscape; the second simulates the types of changes that can happen in evolution.

Methods differ in their predictions for experimentally annotated SAVs, as well as for *in silico* assays of complete mutagenesis (19-non native SAVs). For instance, SIFT and SNAP2 predictions differ more for all possible SAVs in human than for variants with effect on molecular function from PMD (S1 Table). A similar difference is implied between SIFT and PolyPhen-2 [34]. Although the differences amount to “just” 3–8 percentage points, they imply prediction differences for millions of variants. Why do the predictions of the two methods agree more for experimental annotations than for all possible variants?

Assume that the existing methods converged toward the same solution for known data due to the lack of diversity in the training data, *i*.*e*. the same data enforces the same lesson. Put differently, the experimental data focuses on some particular type of effect (that might be easier to predict than the types that remain unknown). This assumption would explain our findings but it seems incorrect. Firstly, methods have not used the exact same type of data: some focus on molecular function, others on disease-causing variants. Secondly, prediction agreement between methods is not higher for strong-impact, disease-causing variants from OMIM than for the neutral and molecular function effect variants from PMD, although stronger variants are predicted better [17,30]. Thirdly, additional recent tests confirm the important differences in predictions for larger data sets, where methods tend to agree more for some observed human variants and less so for others. Thus, the agreement between methods for experimentally annotated data sets is not explained by the assumption that they learned the same from the restricted data.

Could it be that we already have an experimental record for most effect variants? If true, the observed method correlation would be explained. For OMIM, this completeness assumption might not be too far from the truth: It has been argued that through recent advances in deep sequencing the majority of disease-causing variants, in particular in coding regions which are tractable through whole exome sequencing, have already been observed and many are to follow in the near future [35]. However, large-scale *in silico* mutagenesis strongly suggests that many effect variants remain experimentally uncharacterized. If true, the method agreement for experimental annotations would not be explained.

Alternatively, differences between *in silico* mutagenesis predictions and experimental annotations might originate from the bias in the experimental data. Many reasons would explain such a bias. Firstly, the *in vitro* assays may not capture all interactions and constraints under which proteins exist *in vivo*. Secondly, the experimental thresholds for the degree of functional impact (*e*.*g*. change in ∆∆G of binding) required to report a variant as “effect” or “neutral” are subjective. Computational methods will likely zoom into the most consistent data, *i*.*e*. the strongest or simplest effects. Bias might also be introduced by the difficulty in relating the molecular to the system level, *e*.*g*. not every variant that has a high effect on molecular function challenges the organism. Conversely, not every disease is caused by a single SAV. On the contrary, most diseases are likely caused by much more complex mechanisms than single variants. For example, in cancer many variants may affect molecular function; some of these “drive” the cancerous growth, others simply piggyback (passenger mutations). The two have very different biological traits and can be distinguished *in silico* [36]. Nevertheless, the gain from molecular functional effect predictions for describing odds in prognosis is still limited [37].

Finally, the methods’ high agreement might originate from the codon usage. While there is no comprehensive explanation that convincingly maps the codon usage to the biophysical features of the encoded amino acids, there are some preferences built into one of the three bases [38]. SNV-possible variants might therefore tend to alter the biophysical features of an amino acid less than other substitutions. Methods such as SNAP2 are trained to consider variants that maintain the biophysical environment of a residue to be more neutral than others. Hence, SNV-possible might be predicted as more neutral than amino acid substitutions that required more than one nucleotide change. However, since most experimental annotations report effect SAVs, the codon usage correlations are unlikely to help explain the agreement.

### Capturing phenotype effects through molecular function predictions?

In order to bridge the gap from effect upon single protein to effect upon organism, we clearly also have to consider the interaction context of a protein. For instance, predicted effects upon molecular function are much more likely to imply effects upon the organism if the protein is a key player in a crucial pathway than if the protein is “just” a structural protein. Indeed, OMIM SAVs may be so damaging because they preferentially hit crucial proteins. OMIM SAVs constitute one link between molecular effect and disease, albeit possibly an exceptional one. PolyPhen-2 and SNAP2 trained on such disease-effects. The fact that they predict those very well, therefore, is not very meaningful. However, when we retrained a version of SNAP2 without any disease- or system-level related SAVs, we could still predict OMIM SAVs very well (Fig 1). Thus, we established one link between molecular and organism effect.

How could we bridge the gap from the molecular level to that of the organism more efficiently for a larger set of SAVs? As already mentioned: we might succeed by including more relevant knowledge related to interactions. However, success toward this end remains incomplete for the time being. Alternatively, we might consider the integration of gene prioritization tools. These integrate additional orthogonal data such as expression patterns, subcellular localization, information from literature or otherwise manually curated annotations [39,40]. For example, recent work has seen the development of a model to distinguish loss-of-function genes in human, based on conservation and protein interaction data [41]. This however is based on variants that lead to a complete loss of the transcript and therefore not comparable to the SAV effect prediction by SNAP2.

Another idea is to move from the level of SAVs to that of correlated variants [8,23]. This remains challenging: no method can yet predict the effect for all possible pairs of SAVs in all human proteins. However, even for the proteins for which some methods can achieve this: such a refinement might contribute much toward increasing the agreement between computational and experimental deep mutagenesis studies. However, it might contribute little for better bridging the micro and macro level.

### Conclusion

We have presented evidence that methods optimized for predicting the effects of SAVs upon molecular function, such as SNAP2, capture the type of strong effect that leads to monogenic diseases. This was sustained even when excluding disease-causing SAVs from training. Possibly, OMIM-like means “effect upon molecular function strong enough to not have to consider anything else”. We also showed that Mendelian disease-causing SAVs in animals from OMIA (mostly dog and cattle) were predicted even more successfully than those from OMIM. Both these results (OMIM higher than training data although not used, OMIA even higher) imply that methods not focused on phenotype level effects, can capture the strong underlying functional effect signal. OMIM-like SAVs often hit the most conserved position, but a trivial prediction solely based on this conservation fell much behind the level of performance reached by methods such as SNAP2 or PolyPhen-2. Generally, computational and experimental analyses of molecular effects of SAVs cannot explain the effects upon the organism. The integration of gene prioritization and the incorporation of additional data from interactions might contribute to bridging this gap.

## Materials and Methods

### Collecting OMIA variants

We annotated sequence variants in animals using the SQL dump of OMIA (release 08/2015) [24]. Gene symbols and the text from the section *Molecular basis* were extracted for all diseases (i) considered as defect by OMIA and (ii) with the causal variant known. We then read the text and publications to extract variant annotations in the standard format of, *e*.*g*. A11W: native alanine (A) at residue position 11 mutated to tryptophan (W). OMIA already contained 82 variants in this format possibly enabling automated extraction through a regular expression. However, at least one of the 82 was outdated; this fact was mentioned in the description, but would have been missed by automation. Our effort yielded another 96 variants. Thus, we could use 178 OMIA variants in total. Next, we retrieved the protein sequences of the OMIA variants by querying UniProtKB (release 2015_08) with the gene symbol and NCBI taxonomy identifier extracted from OMIA. When we had multiple matches, we chose the top match. Among the 178 variants, three synonymous variants were excluded. Of the remaining 175, 12 had to be excluded because the above protocol did not yield a sequence. In 46 cases a sequence could be retrieved but the amino acid found at the position denoted by OMIA was not the one found in the sequence at that position, *e*.*g*. for OMIA variant A11W, the amino acid at position 11 in the sequence was not alanine (A). In 110 cases the amino acid was found as expected and in seven additional cases shifting the position by +1 yielded the expected sequence. The “+1” accounts for sequences stored without the initiator methionine. Our final data set of 117 variants from 99 sequences (S2 Table) is available at https://rostlab.org/resources/omia. The attrition rate leading to the 117 mutations is summarized again in S3 Table. Most of the variants in the final dataset were from dogs (39%) and cattle (21%). These ratios were comparable to those for original 178 variants (44% and 21%). We annotated another 12 positions with single amino acid deletions and 48 variants leading to premature stop codons. However, since SNAP2 only predicts effect for changes of amino acids not their removal or premature stop of the amino acid sequence, these were not used in the further analysis.

### OMIM, SNPdbe, and PMD

We extracted 5,661 OMIM [5] variants with sequences from SNPdbe [42]. SNAP2 [16] was trained on SAVs from PMD, the Protein Mutation Database [43] as well as human disease variants from OMIM and HumVar [5,28]. For the sets shown in Fig 1A, we trained a version of SNAP2 on only molecular effect variants, *i*.*e*. without variants from OMIM or HumVar, and show cross-validation results for that (TrEffect and TrNeutral). In all other cases, the training set of SNAP2 also included disease variants [16].

### Ortholog mapping for OMIM variants to mouse

Human homologs of the animal genes from OMIM were retrieved using the Biomart interface [44] of Ensembl Genes 82 (release 09/2015) [45]. 271 sequences from the OMIM mutation set were removed because they were not found in the Ensembl set. The remaining 1,293 sequence pairs were aligned using the global alignment implemented in BioPython’s *globalds* with BLOSUM62 as substitution matrix, gap open -10 and gap extend -0.5 [46]. Variants at positions with insertions (aligned against a gap) were removed. After transferring the variants from the human to the mouse sequence, some variants implied no change because for the human X2Y variant, the mouse had Y as its native amino acid, *i*.*e*. the “variant” in mouse would have been a synonymous Y2Y. Removing all such cases and their respective variant in human, the final set comprised 4,229 variants (of the original 5,661 OMIM variants) in both human and the mouse homologs, *i*.*e*. the “*in silico* humanized mouse model” (denoted as “OMIM in mouse” in Fig 1).

### Prediction methods

For all variants, effects were predicted by SIFT [27,47], PolyPhen-2 [12] and SNAP2 [16]. We used SNAP2 with the parameter *tolerate*, that performs predictions even if underlying methods fail, to obtain results for all variants. For some analyses (S2 and S3 Figs, S5 and S7 Figs), we used SNAP2 without alignments as input, by using the *skip* parameter. SIFT predictions were obtained locally with version 4.0.3b [47]. PolyPhen-2 predictions were obtained locally using version 2.2.2 [12]. All three methods used a BLAST database created by merging PDB and UniProtKB (release 2015_08), followed by a redundancy reduction at 80% sequence identity with CD-HIT [48,49]. We used the default cutoffs of each method to obtain binary predictions into either effect or neutral for every variant.

### Statistics

The background effects for the OMIM data (Figs 1D and S1 and S8) were estimated as follows: At every disease variant position, we mutated to either (i) the amino acid denoted in the disease SAV (*OMIM*, Figs 1D and S8) or (ii) considered one randomly out of the SNV-possible variants, *i*.*e*. mutations to amino acids that could occur by a single nucleotide change (*OMIM_rand*, S1 Fig). This simplification was imposed by the incompleteness in the knowledge of the underlying DNA sequences. We assume that our hack approximation to “all SNV-possible” provides a sufficiently accurate approximation.

For the non-disease positions, we sampled a random set of positions without known disease variants from the same proteins (*NotOMIM*). Non-disease positions were never sampled from the first and last 10 residues of a sequence, since SNAP2 uses an input window size of 21. The predicted effect at the NotOMIM positions was evaluated as before. (i) Either given an OMIM mutation such as I10L, we randomly picked a non-disease position with isoleucine and mutated it to leucine (*NotOMIM*, Figs 1D and S8). (ii) Alternatively, we chose a random SNV-possible variant from non-disease positions (*NotOMIM_rand*, S1 Fig).

For the conserved non-disease positions (*NotOMIM conserved*) we considered only non-disease positions that were at least as conserved as the known disease position. For instance, assume a protein P contains two disease variants X25Y and A100B. Randomly choose one out of all positions other than 25 and 100 in P that is at least as conserved as position 25. Then do the same for position 100 and all other variants in other proteins. Skip, if the disease position is the one most conserved in that protein and there is no other position with an equally high conservation. For the not conserved positions, we accordingly used all positions with conservation lower than that of the OMIM SAV. Conservation was measured through the *information per position* value from PSI-BLAST PSSMs created by querying the OMIM sequences against the 80% redundancy reduced database of UniProtKB and PDB mentioned in the previous section. At each NotOMIM conserved or not conserved position, effects were predicted as outlined above for cases i (*NotOMIM (not) conserved*, Figs 1D and S8) and ii (*NotOMIM_rand (not) conserved*, S1 Fig).

The same was repeated using SNAP2 without alignments as input (S2 and S3 Figs). We also show results for the full set of variants, *e*.*g*. “all @ NotOMIM_rand not conserved” are all SNV-possible mutations at all non-disease positions that are less conserved than the position of the original OMIM SAV. “all @ NOT-OMIM conserved” are all OMIM SAVs at all eligible non-disease positions (S1 and S8 Figs). All analyses were also performed on the OMIA set (S4–S7 Figs).

## Supporting Information

S1 Fig SNAP2 predictions towards random SNV-possible variants at different positions in the OMIM set. Analogous to Fig 1D of the main paper but mutating positions to random SNV-possible variants instead of using the OMIM SAV. “OMIM” is repeated from Fig 1A as reference. The numbers above bars give the number of SAVs in the set. Sets prefixed with “all @” contain all possible mutations in the respective set, instead of a random sample.
(TIF)

S2 Fig SNAP2 predictions without alignment input at different positions in the OMIM set. Analogous to Fig 1D of the main paper but using SNAP2 without alignments input. “OMIM using alignments” is repeated from Fig 1A as a reference. The numbers above bars give the number of SAVs in the set. Sets prefixed with “all @” contain all possible mutations in the respective set, instead of a random sample.
(TIF)

S3 Fig SNAP2 predictions without alignment input and towards random SNV-possible variants at different positions in the OMIM set. Analogous to Fig 1D of the main paper but mutating positions to random SNV-possible amino acids instead of using the OMIM SAV. Additionally, SNAP2 is used without alignment input. “OMIM using alignments” is repeated from Fig 1A as a reference. The numbers above bars give the number of SAVs in the set. Sets prefixed with “all @” contain all possible mutations in the respective set, instead of a random sample.
(TIF)

S4 Fig SNAP2 predictions at different positions in the OMIA set. Analogous to Fig 1D of the main paper but on the OMIA set. “OMIA” is repeated from Fig 1B as a reference. The numbers above bars give the number of SAVs in the set. Sets prefixed with “all @” contain all possible mutations in the respective set, instead of a random sample.
(TIF)

S5 Fig SNAP2 predictions without alignment input at different positions in the OMIA set. Analogous to Fig 1D of the main paper but using SNAP2 without alignments input and on the OMIA set. “OMIA using alignments” is repeated from Fig 1B as a reference. The numbers above bars give the number of SAVs in the set. Sets prefixed with “all @” contain all possible mutations in the respective set, instead of a random sample.
(TIF)

S6 Fig SNAP2 predictions towards random SNV-possible variants at different positions in the OMIA set. Analogous to Fig 1D of the main paper but using OMIA and mutating positions to random SNV-possible variants instead of using the OMIA SAV. “OMIA” is repeated from Fig 1B as reference. The numbers above bars give the number of SAVs in the set. Sets prefixed with “all @” contain all possible mutations in the respective set, instead of a random sample.
(TIF)

S7 Fig SNAP2 predictions without alignment input and towards random SNV-possible variants at different positions in the OMIA set. Analogous to Fig 1D of the main paper but using OMIA and mutating positions to random SNV-possible amino acids instead of using the OMIA SAV. Additionally, SNAP2 is used without alignment input. “OMIA using alignments” is repeated from Fig 1B as a reference. The numbers above bars give the number of SAVs in the set. Sets prefixed with “all @” contain all possible mutations in the respective set, instead of a random sample.
(TIF)

S8 Fig SNAP2 predictions at different positions in the OMIM set. Analogous to Fig 1D of the main paper. “OMIM” is repeated from Fig 1A as a reference. The numbers above bars give the number of SAVs in the set. Sets prefixed with “all @” contain all possible mutations in the respective set, instead of a random sample.
(TIF)

S1 Table Pairwise agreement of effect prediction. Shown is the percentage of entries in the respective dataset for which the two given methods agree in binary prediction, *i*.*e*. both predict a neutral or effect variation.
(DOC)

S2 Table The set of 117 OMIA mutations. The 117 mutation extracted by manual review from the OMIA database. Shown are only entries for which a sequence could be found and the mutation mapped onto the sequence (*cf*. S3 Table). All diseases are considered a defect by OMIA annotation. Organism shows the NCBI taxonomy id. Variants marked with *, are those where the position was shifted one forward (Methods, S3 Table). The full set including the sequences is also available at rostlab.org/resources/omia.
(DOC)

S3 Table Attrition rate of OMIA annotations. AA deletion describes cases where a single amino acid is deleted without affecting the reading frame. Nonsense are mutations to a premature stop codon. These two cases were extracted from OMIA but not used in the analysis. For the amino acid substitution set *No seq*. describes that no sequence was found for the given combination of taxonomy id and gene id (Methods). *No match* describes that a sequence was found but the amino acid at the position given by OMIA was not the one expected from the annotated mutation. *Match* are all cases where this was the case, and *Match+1* were the amino acid fit after shifting one position to the right. Highlighted in green are the cases forming the final set of 117 mutations used for the analysis.
(DOC)
