# Supplementary figures and images for: Predicted Molecular Effects of Sequence Variants Link to System Level of Disease
Source: PLoS Comput Biol. 2016 Aug 18;12(8):e1005047. doi: 10.1371/journal.pcbi.1005047 (PMC4990455; doi:10.1371/journal.pcbi.1005047)

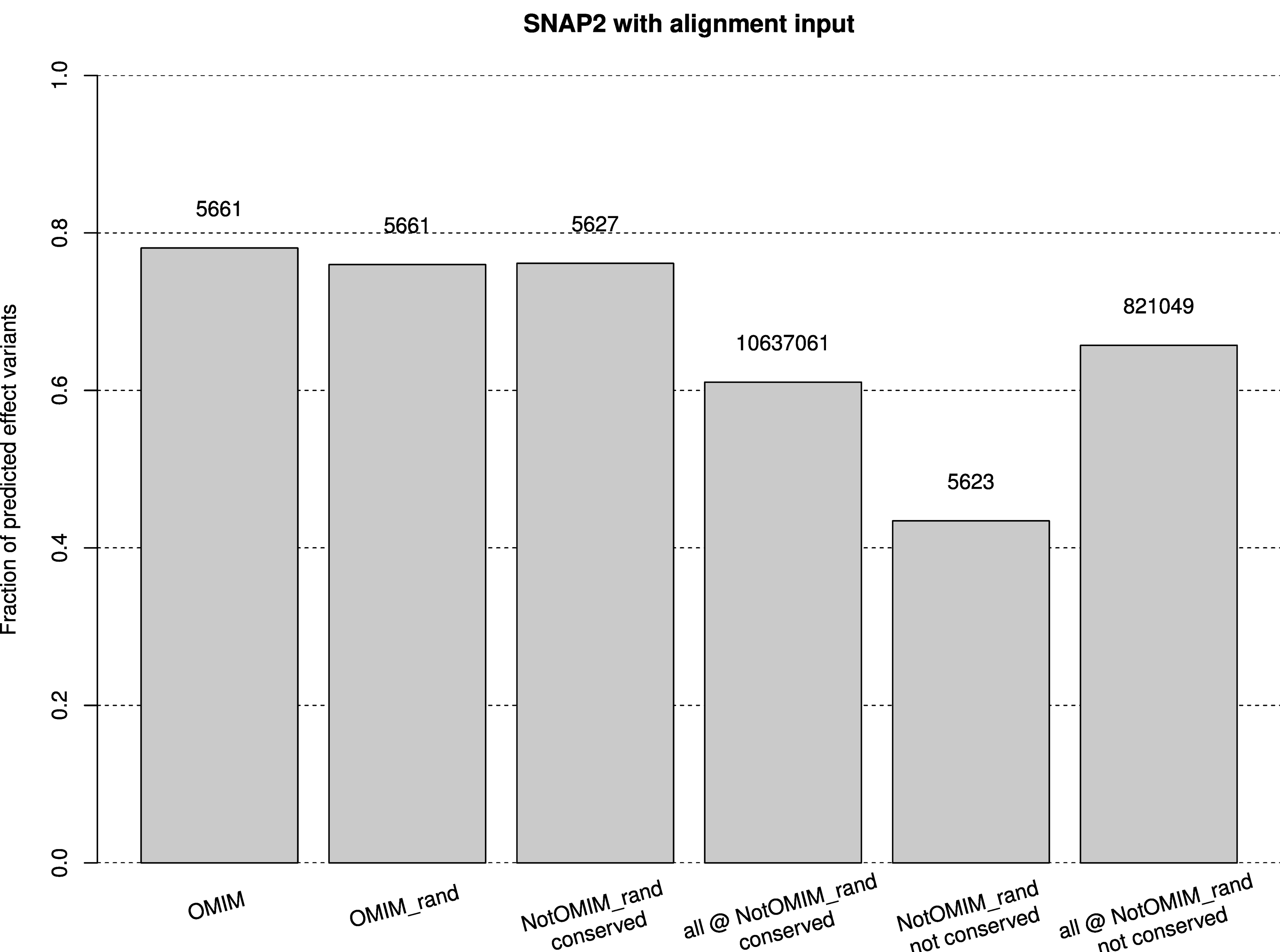

Supplement: S1 Fig — Analogous to Fig 1D of the main paper but mutating positions to random SNV-possible variants instead of using the OMIM SAV. “OMIM” is repeated from Fig 1A as reference. The numbers above bars give the number of SAVs in the set. Sets prefixed with “all @” contain all possible mutations in the respective set, instead of a random sample. (TIF) [file pcbi.1005047.s001.tif]

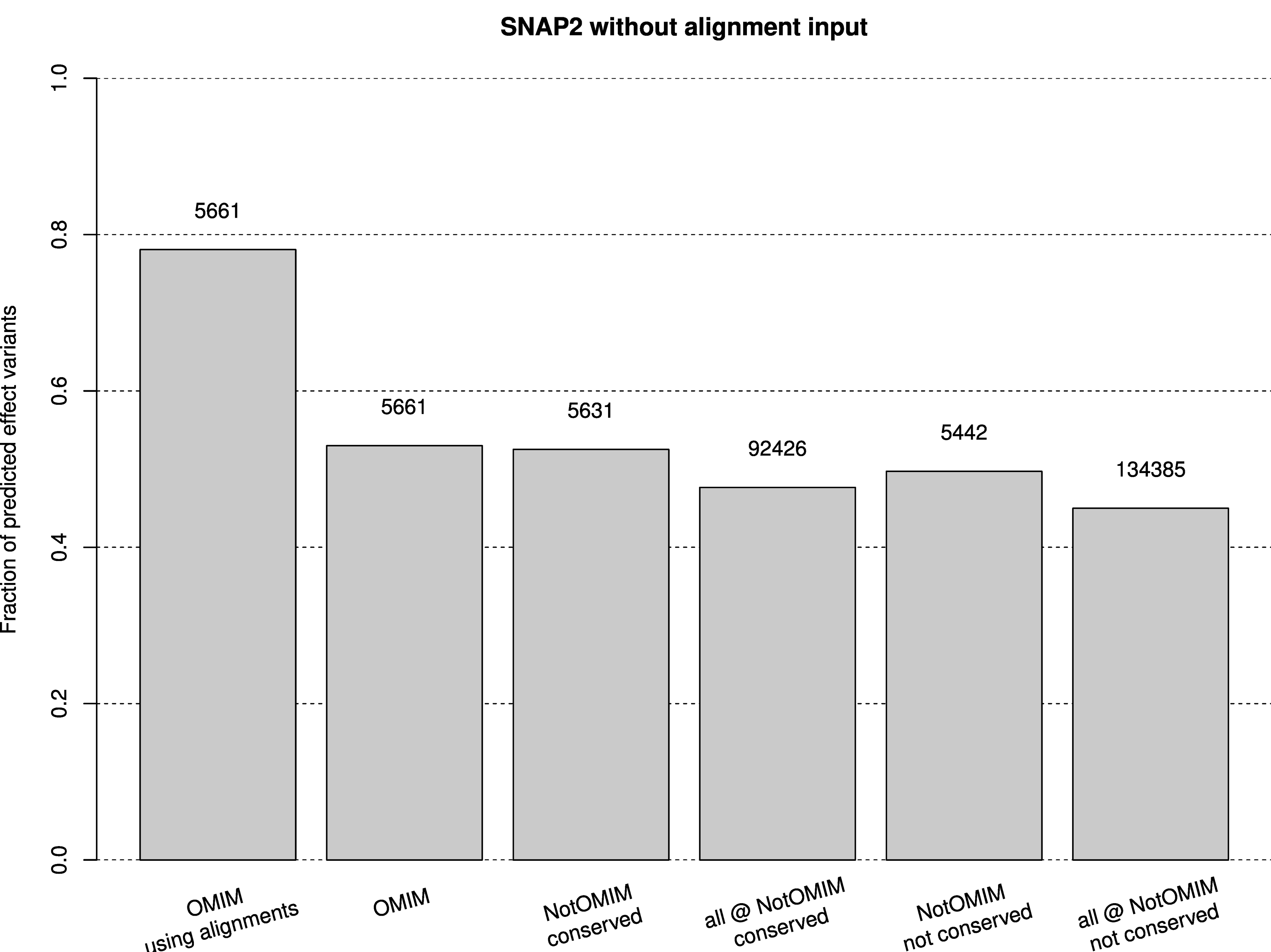

Supplement: S2 Fig — Analogous to Fig 1D of the main paper but using SNAP2 without alignments input. “OMIM using alignments” is repeated from Fig 1A as a reference. The numbers above bars give the number of SAVs in the set. Sets prefixed with “all @” contain all possible mutations in the respective set, instead of a random sample. (TIF) [file pcbi.1005047.s002.tif]

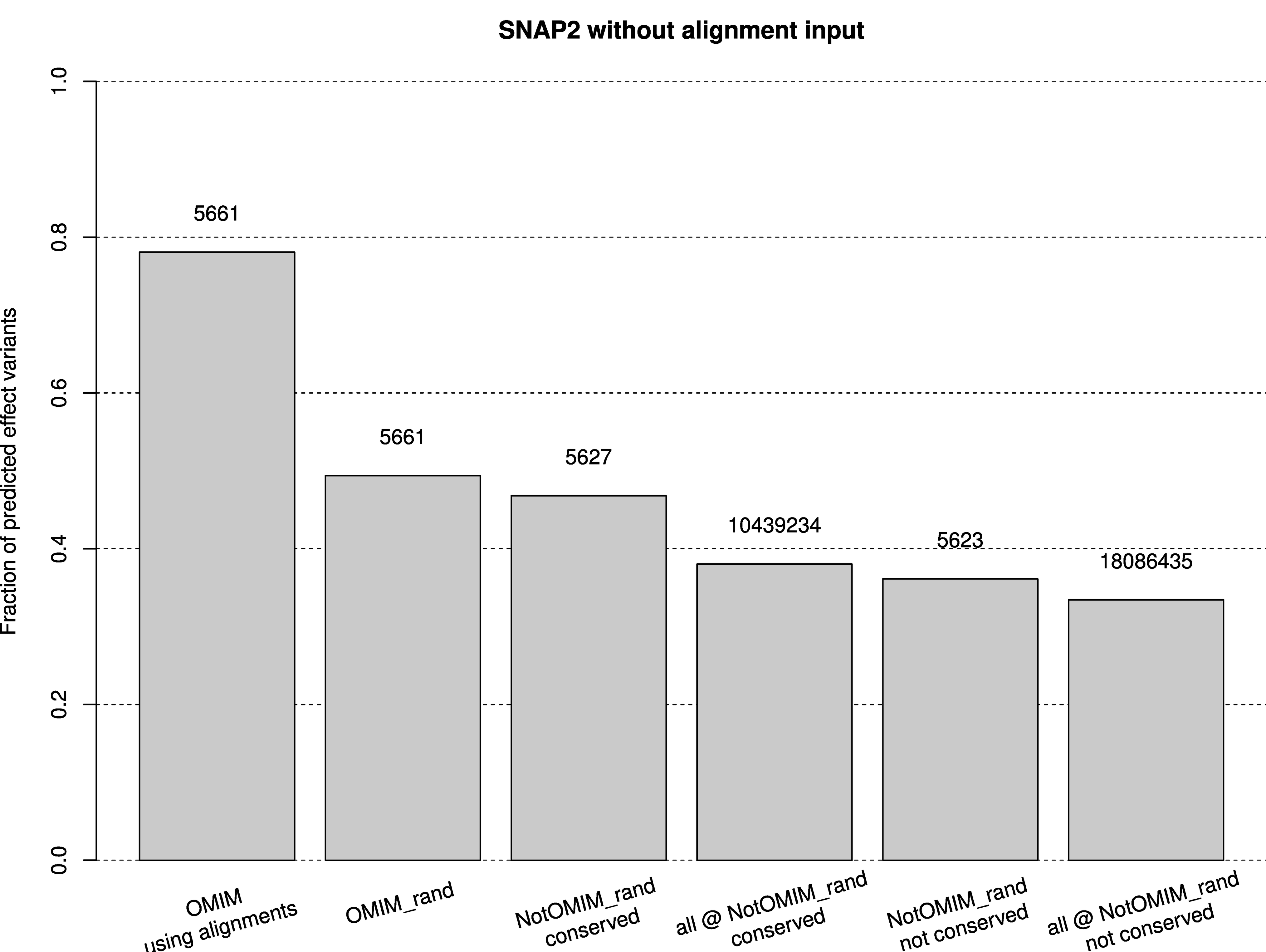

Supplement: S3 Fig — Analogous to Fig 1D of the main paper but mutating positions to random SNV-possible amino acids instead of using the OMIM SAV. Additionally, SNAP2 is used without alignment input. “OMIM using alignments” is repeated from Fig 1A as a reference. The numbers above bars give the number of SAVs in the set. Sets prefixed with “all @” contain all possible mutations in the respective set, instead of a random sample. (TIF) [file pcbi.1005047.s003.tif]

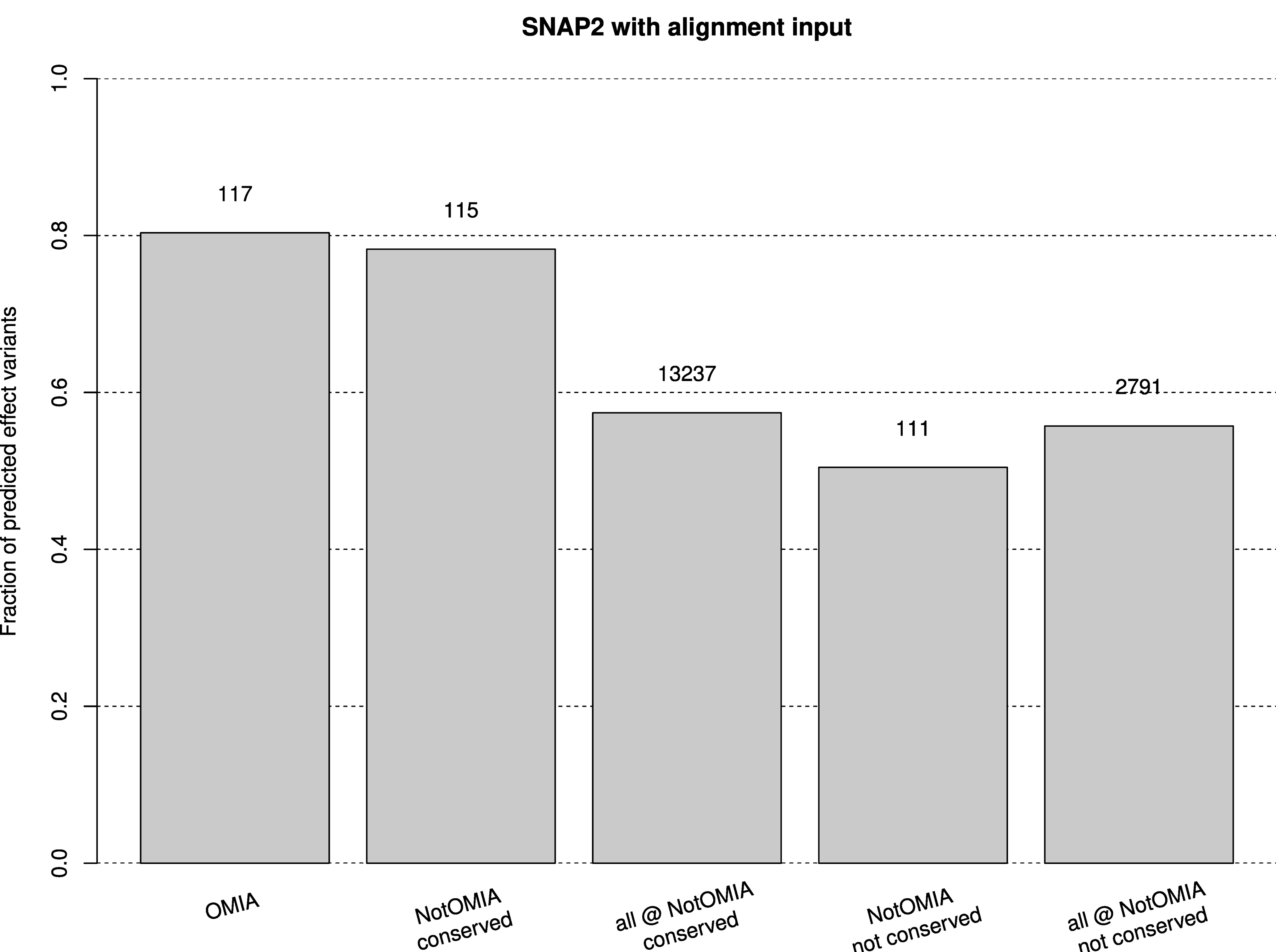

Supplement: S4 Fig — Analogous to Fig 1D of the main paper but on the OMIA set. “OMIA” is repeated from Fig 1B as a reference. The numbers above bars give the number of SAVs in the set. Sets prefixed with “all @” contain all possible mutations in the respective set, instead of a random sample. (TIF) [file pcbi.1005047.s004.tif]

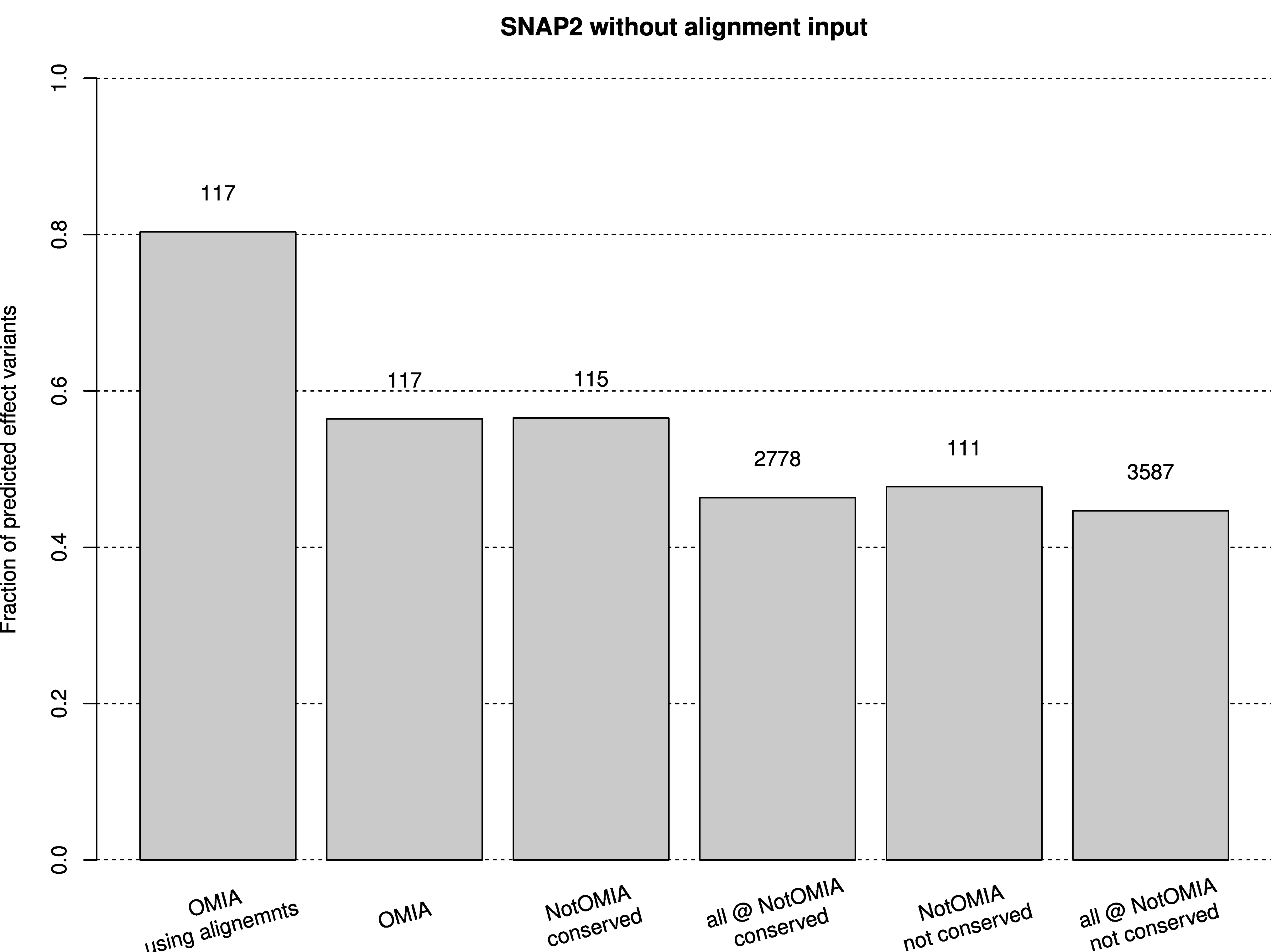

Supplement: S5 Fig — Analogous to Fig 1D of the main paper but using SNAP2 without alignments input and on the OMIA set. “OMIA using alignments” is repeated from Fig 1B as a reference. The numbers above bars give the number of SAVs in the set. Sets prefixed with “all @” contain all possible mutations in the respective set, instead of a random sample. (TIF) [file pcbi.1005047.s005.tif]

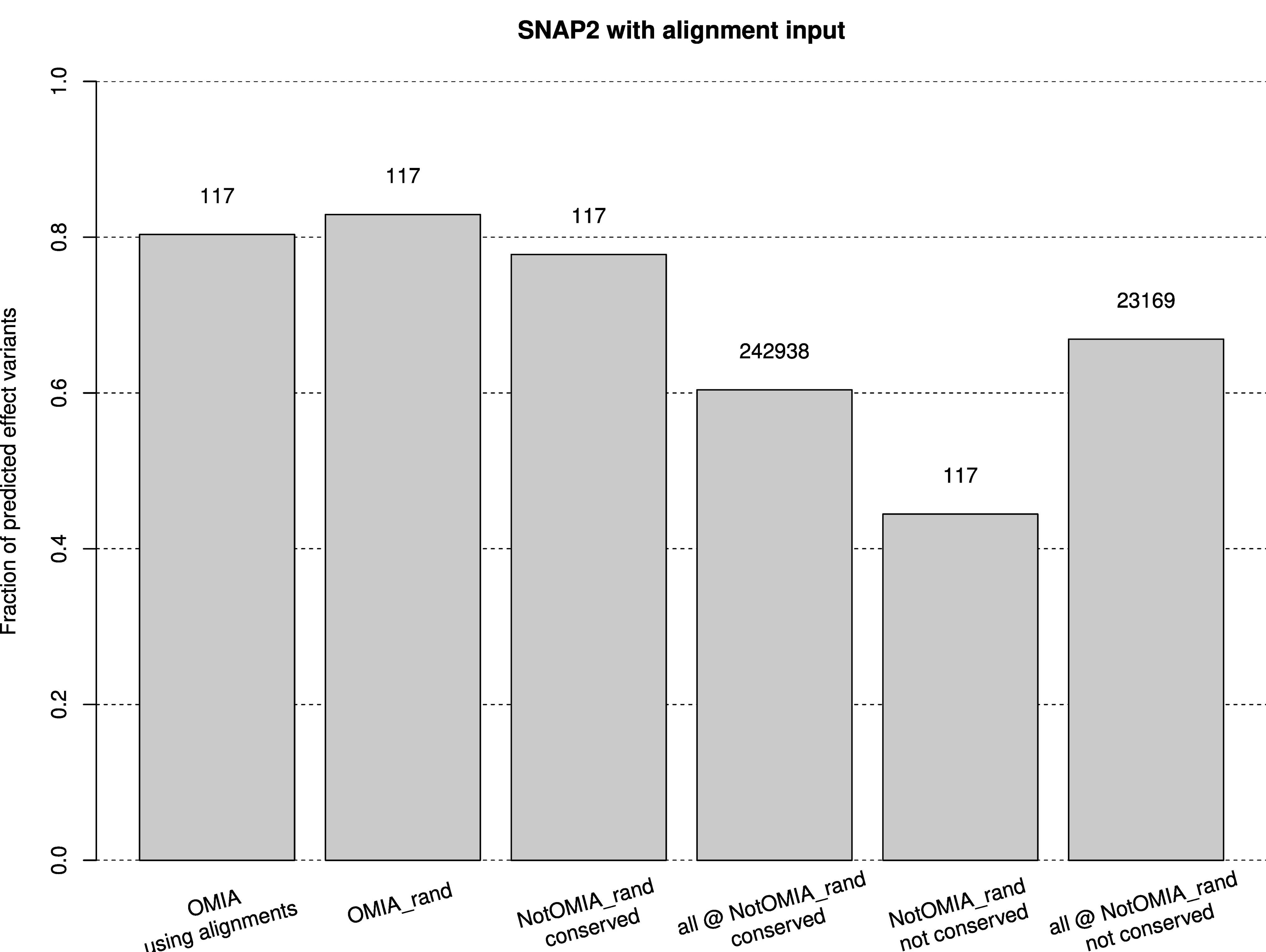

Supplement: S6 Fig — Analogous to Fig 1D of the main paper but using OMIA and mutating positions to random SNV-possible variants instead of using the OMIA SAV. “OMIA” is repeated from Fig 1B as reference. The numbers above bars give the number of SAVs in the set. Sets prefixed with “all @” contain all possible mutations in the respective set, instead of a random sample. (TIF) [file pcbi.1005047.s006.tif]

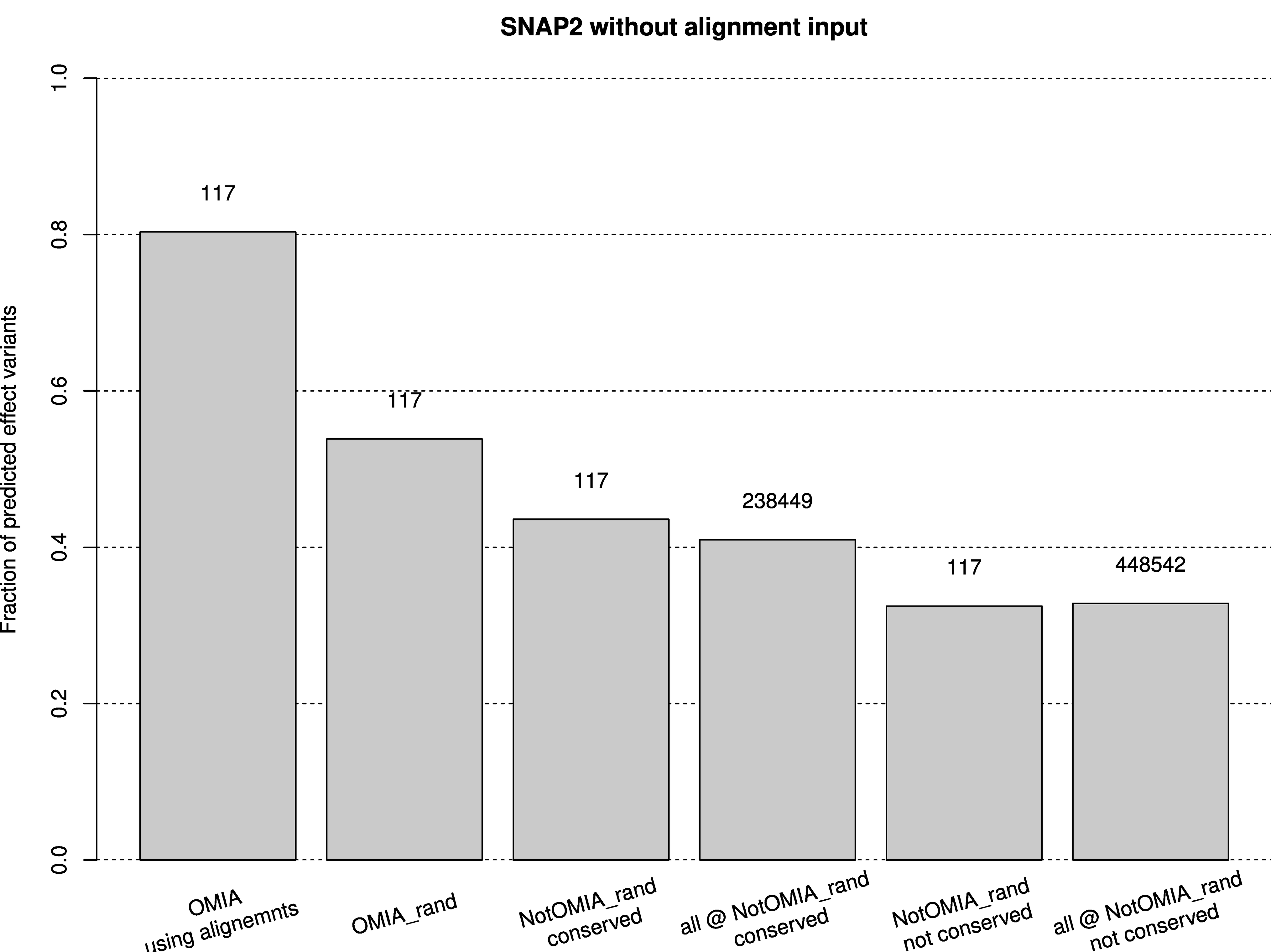

Supplement: S7 Fig — Analogous to Fig 1D of the main paper but using OMIA and mutating positions to random SNV-possible amino acids instead of using the OMIA SAV. Additionally, SNAP2 is used without alignment input. “OMIA using alignments” is repeated from Fig 1B as a reference. The numbers above bars give the number of SAVs in the set. Sets prefixed with “all @” contain all possible mutations in the respective set, instead of a random sample. (TIF) [file pcbi.1005047.s007.tif]

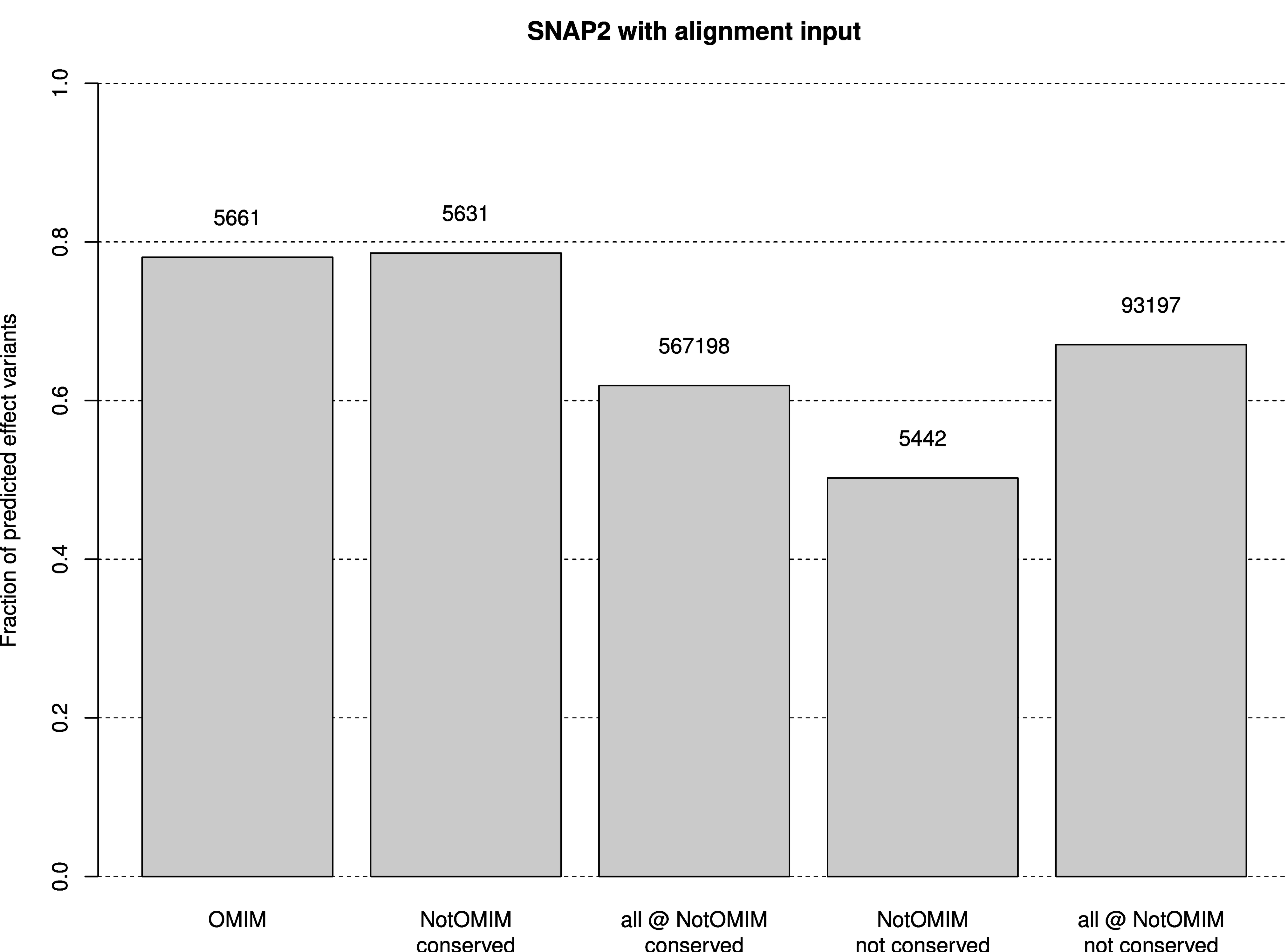

Supplement: S8 Fig — Analogous to Fig 1D of the main paper. “OMIM” is repeated from Fig 1A as a reference. The numbers above bars give the number of SAVs in the set. Sets prefixed with “all @” contain all possible mutations in the respective set, instead of a random sample. (TIF) [file pcbi.1005047.s008.tif]
