# Supplementary material for: Predicted Molecular Effects of Sequence Variants Link to System Level of Disease
Source: PLoS Comput Biol. 2016 Aug 18;12(8):e1005047. doi: 10.1371/journal.pcbi.1005047 (PMC4990455; doi:10.1371/journal.pcbi.1005047)
Supplement: S1 Table — Shown is the percentage of entries in the respective dataset for which the two given methods agree in binary prediction, i.e. both predict a neutral or effect variation. (DOC) [file pcbi.1005047.s009.doc]

Table S1: Pairwise agreement of effect prediction.

Shown is the percentage of entries in the respective dataset for which two given methods agree in binary prediction, *i.e.* both predict a neutral or effect variation.

|  | **Human, 19-non-native** | **PMD** | **OMIM** | **OMIA** |
| --- | --- | --- | --- | --- |
| *∆(SNAP-SIFT)* | 79 | 82.2 | 82 | 90 |
| *∆(SNAP-PolyPhen2)* |  | 76.2 |  |  |
| *∆(SIFT-PolyPhen2)* | 69 [1] | 78.4 |  |  |

[1]: Liu X, Jian X, Boerwinkle E (2013) dbNSFP v2.0: a database of human non-synonymous SNVs and their functional predictions and annotations. Hum Mutat 34: E2393-2402.
