# Supplementary material for: Predicted Molecular Effects of Sequence Variants Link to System Level of Disease
Source: PLoS Comput Biol. 2016 Aug 18;12(8):e1005047. doi: 10.1371/journal.pcbi.1005047 (PMC4990455; doi:10.1371/journal.pcbi.1005047)
Supplement: S2 Table — The 117 mutation extracted by manual review from the OMIA database. Shown are only entries for which a sequence could be found and the mutation mapped onto the sequence (cf. S3 Table). All diseases are considered a defect by OMIA annotation. Organism shows the NCBI taxonomy id. Variants marked with *, are those where the position was shifted one forward (Methods, S3 Table). The full set including the sequences is also available at rostlab.org/resources/omia. (DOC) [file pcbi.1005047.s010.doc]

Table S1: The set of 117 OMIA mutations.

The 117 mutation extracted by manual review from the OMIA database. Shown are only entries for which a sequence could be found and the mutation mapped onto the sequence (cf. Table S3). All diseases are considered a defect by OMIA annotation. Organism shows the NCBI taxonomy id. Variants marked with *, are those where the position was shifted one forward (Methods, Table S3). The full set including the sequences is also available at rostlab.org/resources/omia.

| **OMIA ID** | **Organism** | **OMIA Gene** | **UniProtAC** | **Variant** |
| --- | --- | --- | --- | --- |
| 2547 | 10141 | Man2b1 | Q8VHC8 | R227W |
| 365 | 9913 | LYST | Q9TTK4 | H2015R |
| 1204 | 9913 | FBN1 | P98133 | E1200K |
| 1280 | 9913 | MSTN | O18836 | C313Y |
| 1280 | 9913 | MSTN | O18836 | L64P |
| 608 | 9913 | KRT5 | Q5XQN5 | E478K |
| 3053 | 9913 | PMEL | Q06154 | A612E |
| 1162 | 9913 | ITGB2 | P32592 | D128G |
| 20 | 9913 | MAN2B1 | Q29451 | F321L |
| 20 | 9913 | MAN2B1 | Q29451 | R221H |
| 1891 | 9913 | PYGM | P79334 | R490W* |
| 3321 | 9913 | MITF | F1MCS8 | R210I |
| 2903 | 9913 | GH1 | P01246 | T200M |
| 1634 | 9913 | KDSR | Q2KIJ5 | A175T |
| 2387 | 9913 | RASGRP2 | A6N9I4 | L234P |
| 2857 | 9913 | ATP2A1 | Q0VCY0 | R559C |
| 2880 | 9913 | ATP2A1 | Q0VCY0 | R164H |
| 2880 | 9913 | ATP2A1 | Q0VCY0 | G211V |
| 2880 | 9913 | ATP2A1 | Q0VCY0 | G285V* |
| 2665 | 9913 | SLC35A3 | Q6YC49 | V180F |
| 2219 | 9913 | SPAST | A2VDN5 | R560Q |
| 2858 | 9913 | SLC6A5 | F1N5R0 | L270P |
| 2899 | 9913 | F8 | G5E5W1 | L2153H |
| 3557 | 9913 | GART | Q59A32 | N290T |
| 3719 | 9913 | COL2A1 | P02459 | G960R |
| 3542 | 9913 | IARS | A7MBC5 | V79L |
| 3770 | 9913 | SUGT1 | Q2KIK0 | W317R |
| 2669 | 9913 | NHLRC2 | A4IF69 | V311A |
| 3555 | 9913 | SMC2 | F1MY41 | F1135S |
| 3387 | 9913 | KDM2B | E1BLZ5 | D835N |
| 2869 | 9913 | NAGLU | A6QM01 | E452K |
| 2650 | 9615 | VWF | Q28295 | N1646S |
| 1311 | 9615 | HCRTR2 | Q9TUP7 | E54K |
| 2071 | 9615 | COL7A1 | F1PIP7 | G1906S |
| 2648 | 9615 | CAT | O97492 | A327T |
| 3314 | 9615 | CNGB3 | Q8MJD7 | D262N |
| 506 | 9615 | SOD1 | Q8WNN6 | E40K |
| 506 | 9615 | SOD1 | Q8WNN6 | T18S |
| 2053 | 9615 | CLCN1 | Q9MZT1 | T268M |
| 1163 | 9615 | ITGB2 | F1Q1H6 | C36S |
| 1728 | 9615 | ITGA2B | F1PRU9 | D399H* |
| 1258 | 9615 | GUSB | O18835 | R166H |
| 1258 | 9615 | GUSB | O18835 | P289L |
| 729 | 9615 | PFKM | P52784 | R184W |
| 702 | 9615 | GLB1 | Q9TRY9 | R60H |
| 1140 | 9615 | GALC | P54804 | Y158S |
| 1515 | 9615 | PKLR | Q29536 | V283A |
| 1515 | 9615 | PKLR | Q29536 | G332S |
| 2975 | 9615 | CTSD | Q4LAL9 | M199I |
| 2977 | 9615 | CLN8 | Q5JZQ7 | L164P |
| 2673 | 9615 | RHO | P32308 | T4R |
| 723 | 9615 | G6PC | O19133 | M121I |
| 2922 | 9615 | SERPINH1 | E2RHY7 | L326P |
| 2885 | 9615 | DNM1 | F1PAK1 | R256L |
| 1595 | 9615 | PLP1 | P23294 | H37P |
| 2973 | 9615 | ARSG | Q32KH9 | R99H |
| 1384 | 9615 | COL1A2 | O46392 | G208A |
| 1484 | 9615 | KLKB1 | F1PNV5 | F330I |
| 2900 | 9615 | ATF2 | F1PCQ4 | M51R |
| 2635 | 9615 | FLCN | B1NLQ8 | H255R |
| 2451 | 9615 | NDRG1 | E2RMB9 | G98V |
| 3065 | 9615 | BEST1 | A5H7G8 | G161D |
| 3066 | 9615 | BEST1 | A5H7G8 | G489V |
| 2182 | 9615 | JAK2 | E2RPW6 | V617F |
| 2182 | 9615 | JAK2 | E2RPW6 | C618L |
| 2980 | 9615 | ADAMTSL2 | F1PBN7 | R221C |
| 3097 | 9615 | CLN6 | Q5JZQ8 | W277R |
| 2979 | 9615 | MTM1 | F1Q1K0 | N156K* |
| 2661 | 9615 | CYTB | Q34101 | V98M |
| 2489 | 9615 | PRCD | Q00LT9 | C2Y |
| 3301 | 9615 | LOC607355 | J9NU87 | G102S |
| 3348 | 9615 | SEL1L | F1PVX5 | S658P |
| 3737 | 9615 | GNAS | P63091 | R201C |
| 3737 | 9615 | GNAS | P63091 | R201H |
| 3737 | 9615 | GNAS | P63091 | R201S |
| 3737 | 9615 | GNAS | P63091 | R201L |
| 3737 | 9615 | GNAS | P63091 | Q227H |
| 3737 | 9615 | GNAS | P63091 | Q227R |
| 3737 | 9615 | GNAS | P63091 | L203P |
| 2984 | 9615 | BRAF | F1P873 | V595E |
| 2730 | 9615 | SUV39H2 | E2RHJ2 | N324K |
| 3699 | 9615 | RAB24 | E2R4M1 | Q38P |
| 3471 | 9615 | COL11A2 | F1PQD5 | R48P |
| 3762 | 9615 | ATG4D | E2RDP2 | A430T |
| 3539 | 9615 | HEXA | E2RIM8 | E323K |
| 3703 | 9615 | SLC45A2 | Q2PUG5 | G493D |
| 3628 | 9615 | ADAMTS10 | E2RML2 | G661R |
| 2618 | 9615 | FAM83G | F1PQU4 | R52P |
| 3370 | 8090 | DMRT1 | Q801F8 | C53R |
| 2790 | 9940 | urod | Q8HY31 | L131P |
| 1646 | 9940 | PRNP | P23907 | R171Q |
| 2844 | 9940 | CLN6 | Q1PAG8 | R62C |
| 1236 | 9940 | PITX3 | D3GN34 | R113P |
| 709 | 9940 | GBA | W5P1A5 | C381Y |
| 3365 | 9940 | HEXA | W5QC89 | G444R |
| 120 | 9031 | ABCA1 | E1C2W8 | E89K |
| 1270 | 9031 | WWP1 | Q5F372 | R441Q |
| 2764 | 9031 | FMO6P | Q8QH01 | T329S |
| 3023 | 9031 | MX1 | B2X021 | S631N |
| 1607 | 9031 | SLC45A2 | F1NJ93 | Y277C |
| 1607 | 9031 | SLC45A2 | F1NJ93 | L347M |
| 1207 | 9796 | EDNRB | O62709 | I118K |
| 61 | 9796 | SCN4A | Q28371 | F1416L |
| 2032 | 9796 | GYS1 | B2L109 | R309H |
| 2646 | 9796 | PPIB | A5YBL8 | G39R |
| 3102 | 9796 | SLC5A3 | F6RYW1 | P446L |
| 3326 | 9796 | CLCN1 | F6RP34 | D593A* |
| 3715 | 9796 | ACAN | F7C3C6 | V424M |
| 3549 | 9595 | SLC45A2 | G3QQ65 | G518R |
| 12 | 9986 | PLP1 | P47789 | H37Q* |
| 1342 | 9685 | NPC1 | M3VUX6 | C955S |
| 1257 | 9685 | GUSB | O97524 | E351K |
| 700 | 9685 | GLB1 | O19015 | R483H |
| 2942 | 9685 | HMBS | D3W810 | R149W |
| 2942 | 9685 | HMBS | D3W810 | A84T |
| 2941 | 9685 | UROS | M3WBN3 | S47F |
| 3109 | 9685 | KRT71 | E1AB55 | S439G* |
