# Supplementary material for: Predicted Molecular Effects of Sequence Variants Link to System Level of Disease
Source: PLoS Comput Biol. 2016 Aug 18;12(8):e1005047. doi: 10.1371/journal.pcbi.1005047 (PMC4990455; doi:10.1371/journal.pcbi.1005047)
Supplement: S3 Table — AA deletion describes cases where a single amino acid is deleted without affecting the reading frame. Nonsense are mutations to a premature stop codon. These two cases were extracted from OMIA but not used in the analysis. For the amino acid substitution set No seq. describes that no sequence was found for the given combination of taxonomy id and gene id (Methods). No match describes that a sequence was found but the amino acid at the position given by OMIA was not the one expected from the annotated mutation. Match are all cases where this was the case, and Match+1 were the amino acid fit after shifting one position to the right. Highlighted in green are the cases forming the final set of 117 mutations used for the analysis. (DOC) [file pcbi.1005047.s011.doc]

Table S1: Attrition rate of OMIA annotations.

AA deletion describes cases where a single AA is deleted without affecting the reading frame. Nonsense are mutations to a premature stop codon. These two cases were extracted from OMIA but not used in the analysis. For the AA substitution set *No seq.* describes that no sequence was found for the given combination of taxonomy id and gene id (Methods). *No match* describes that sequence was found but the amino acid at the position given by OMIA was not the one expected from the annotated mutation. *Match* are all cases where this was the case, and *Match+1* were the amino acid fit after shifting one position to the right. Highlighted in green are the cases forming the final set of 117 mutations used for the analysis.

| AA deletion | Nonsense | AA substitution (178) | | | | |
| --- | --- | --- | --- | --- | --- | --- |
| 12 | 48 | Synonymous | Non-synonymous (175) | | | |
|  |  | 3 | No seq. | No match | **Match** | **Match+1** |
|  |  |  | 12 | 46 | **110** | **7** |
